# Chemical Defenses in Medusozoa

**DOI:** 10.3390/md23060229

**Published:** 2025-05-28

**Authors:** Oliver J. Lincoln, Jonathan D. R. Houghton, Muhammad Zakariya, Chiara Lauritano, Isabella D’Ambra

**Affiliations:** 1 School of Biological Sciences, Queen’s University Belfast, 19 Chlorine Gardens, Belfast BT9 5DL, Co. Antrim, UK; olincoln01@qub.ac.uk (O.J.L.); j.houghton@qub.ac.uk (J.D.R.H.); 2 Queen’s University Belfast Marine Laboratory, 12-13 The Strand, Portaferry BT22 1PF, Co. Down, UK; 3 Research Centre for Experimental Marine Biology and Biotechnology, Plentzia Marine Station, University of Basque Country (PiE-EHU/UPV), 48620 Plentzia, Spain; zakariyakhan291@gmail.com; 4 Ecosustainable Marine Biotechnology Department, Stazione Zoologica Anton Dohrn, Via Acton n. 55, 80133 Naples, Italy; chiara.lauritano@szn.it; 5 Integrative Marine Ecology Department, Stazione Zoologica Anton Dohrn, Villa Comunale, 80121 Naples, Italy; 6 National Biodiversity Future Center (NBFC), Piazza Marina 61, 90133 Palermo, Italy

**Keywords:** photoprotection, antimicrobial peptides, antioxidants, endosymbionts, ecology, biotechnology, integrated approaches

## Abstract

Cnidarian defensive strategies are commonly associated with the toxins they synthesize. Because toxins have negative, sometimes lethal, effects on humans, research has focused on them for medical and biotechnological applications. However, Cnidaria possess a variety of defensive systems complementing toxins. In recent decades, ecological and biotechnological studies have shed light on these systems, particularly in Anthozoa, while the knowledge of defensive systems different from toxins has remained limited in Medusozoa (Cubozoa, Hydrozoa, Scyphozoa and Staurozoa). In this review, we collected the scattered information available in the literature and organized it into four main topics: UV-light protection compounds, antioxidants, antimicrobial peptides, and endosymbionts. Within the topics, we found the largest amount of data refers to antimicrobial activities, which suggests this line of research as a potential exploitation of this group of organisms often appearing in large aggregates. We also found that some Medusozoa have been studied in detail as model organisms, although the close phylogenetic relationship among classes suggests that some defensive strategies may be common to other members of different classes. Indeed, an integrated understanding of defensive systems has the potential to inform not only ecological and evolutionary frameworks, but also biotechnological applications—from the identification of novel antioxidants or antimicrobial agents to the valorization of Medusozoan biomass.

## 1. Introduction

Chemical defenses of Cnidaria are often uniquely associated with toxins and venoms secreted by cnidocysts, which are the organelles embedded within the tissues of all Cnidaria and are specialized to produce toxic compounds. Cnidocysts vary remarkably across the 3 sub-phyla of Cnidaria (Anthozoa, Endocnidozoa, Medusozoa [1,2]. Used by Cnidarians to discourage predators and to paralyze prey in order to facilitate capture, toxins are finding various applications in biotechnology fields [3]. However, chemical defenses in Cnidarians are not exclusively linked to cnidocysts and toxins.

**Figure 1:**
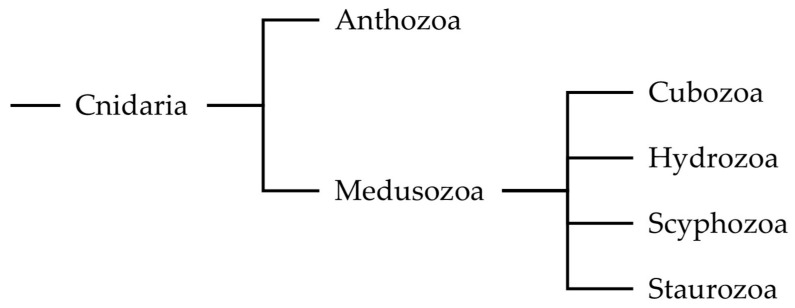
A phylogenetic tree defining Medusozoa and the classes therein for the purposes of this review.

In recent decades, advances in molecular techniques and natural product discovery have shed light on a range of defensive strategies employed by Anthozoa, including antimicrobial peptides [4], and photoprotective compounds [5,6]. Conversely, less studies have been dedicated to Medusozoa (Figure 1) [7]. Nevertheless, these insights have begun to highlight this subphylum’s biochemical and ecological versatility, which remains comparatively underrepresented in the literature relative to Anthozoa.

In this review, ‘chemical defenses’ refers to those compounds reported to be used by Medusozoa for defensive purposes. This includes both endogenously synthesized compounds, compounds assimilated from dietary sources, and compounds synthesized by associated microorganisms. Each of these types of compounds is employed as a potential defense and so will be considered in this review.

Despite the fact that mucus is secreted by all Cnidarians and plays an important role as a defensive strategy within this phylum [8], we have decided not to include this topic in the present review. Mucus secretion is remarkable in many Medusozoa, such as Scyphomedusae, because they attain a large body size, but secretion rates have been determined using different methodologies [9,10,11,12,13], which makes results unfeasible to compare. Studies into mucus composition, which reflects the elemental composition of the producing Medusozoan [11,14,15,16,17,18,19], have confirmed its ecological role within microbial food webs and biogeochemical cycles, but have also suggested its employment as a nanoparticle trap within the marine environment [18,20,21] within the framework of the exploitation of Medusozoa for biotechnological applications [22].

This review is timely in bringing together emerging evidence on Medusozoan defensive biology, particularly in the context of marine natural products and bioactive compound discovery. A more integrated understanding of these systems has the potential to inform not only ecological and evolutionary frameworks, but also biotechnological applications—from the identification of novel antioxidant or antimicrobial agents to the valorization of Medusozoan biomass. By drawing attention to both recent progress and current knowledge gaps, this synthesis aims to support renewed research interest in Medusozoa as a source of biologically and pharmacologically relevant compounds.

## 2. Ultra-Violet Light Protective Compounds

UV light is separated into three categories—UV-A (315–400 nm), UV-B (280–315 nm), and UV-C (100–280 nm), with UV-A having the longest wavelength and UV-C the shortest [23]. The shorter wavelengths are more harmful to biological systems [24], but are also attenuated more rapidly in seawater [25,26]. UV-A can generate singlet oxygen [27] and other reactive oxygen species (ROS) [28], yet also provides energy for photolyase enzymes that repair UV-induced DNA damage [24]. It is also 10 to 100 times more abundant than UV-B in the fraction of UV radiation reaching sea level [29]. UV-B, in contrast, can induce cytotoxic DNA lesions—including both single and double strand breaks [27]—disrupt proteins and pigments [30], and generate ROS in higher volumes than UV-A [28]. UV-C, the most energetic band, is fully absorbed by atmospheric ozone and oxygen [27,31], and thus does not impact marine systems directly.

Photoprotective compounds are therefore important in mitigating the harmful effects of UV radiation in marine organisms. However, while considerable research has focused on such compounds in Anthozoa—particularly in reef-building corals [5,6]—comparable studies in Medusozoa remain limited. Some parallels can be drawn, such as the occurrence of green fluorescent protein (GFP) in both groups [6,32], yet the functional roles of such compounds in Medusozoa remain unclear. Understanding whether similar photoprotective mechanisms are at play represents an important avenue for future investigation.

Of all the Medusozoa, GFP is found only in the Hydrozoa [33]. In corals, fluorescent proteins have been shown to be photoprotective for the zooxanthellate algae, and can prevent bleaching [6]. Since GFP has only been shown to be photoprotective in the presence of a symbiotic alga, it may only be photoprotective for the subset of Hydrozoans that have symbiotic relationships with algae [34,35]. There is not enough evidence to determine if GFP is photoprotective for non-symbiotic species of Hydrozoa, so further research is required.

GFP is not the only photoprotective molecule in Hydrozoa, however. Tridentatols are a group of secondary metabolite compounds isolated from *Tridentata marginata* that were found to strongly absorb both UV-A and UV-B over a wide range of wavelengths [36]. These tridentatols have only been isolated from *Tridentata marginata*, so may not be cosmopolitan across the Hydrozoa. Cassio Blue is a blue pigment found in *Cassiopea xamachana* that, like GFP, was found to be photoprotective for the symbiotic algae [37], as it is found in high concentrations in regions of high algal concentration. However, its relatively low efficiency suggests that its primary role may lie elsewhere—potentially in metal ion regulation [38]—highlighting the need for further investigation into the multifunctionality of pigments in Hydrozoa.

Both of these photoprotective compounds have been identified in shallow-water species of gelatinous zooplankton [39,40]. The elevated UV irradiance in shallow marine environments may exert selective pressure for the development of UV-protective traits. Alternatively, their apparent restriction to shallow-water species could reflect sampling bias, as these organisms are more accessible and logistically easier to study [41]. However, with data currently limited to just two species, it is not possible to meaningfully assess the relative influence of ecological selection versus sampling artifact.

Medusozoans have been observed to engage in diel vertical migration [42], with some species potentially engaging in this migration to avoid UV exposure, descending into deeper, less irradiated waters during daylight hours [43,44,45]. By physically avoiding peak UV conditions, these species may experience reduced selective pressure to evolve or maintain direct molecular photoprotective compounds, such as UV-absorbing pigments. This behavioral strategy could therefore help explain why relatively few such compounds have been identified in Medusozoa to date. While UV avoidance is unlikely to be the sole driver of vertical migration [46,47,48,49], it likely acts alongside other ecological factors to shape this behavior. Notably, Medusozoa possess a diverse array of oxidative stress response systems, suggesting that rather than investing in primary photoprotection, their evolutionary trajectory may have favored secondary defenses—such as reactive oxygen species neutralization and DNA repair mechanisms—to mitigate UV-induced damage.

## 3. Defense Against Reactive Oxygen Species

### 3.1. Production of Reactive Oxygen Species

ROS can be synthesized within biological systems in multiple ways. In Eukaryotes, the mitochondrial electron transport chain is the major source of both superoxide anions (O_2_^−^) and hydrogen peroxide (H_2_O_2_), although other enzymes and UV radiation, play a part in their generation too, as well as of other ROS such as hydroxyl radicals (OH^−^) and singlet oxygen (^1^O_2_^−^) [50]. For some Cnidaria with endosymbiotic algae, ROS are also produced by photosynthesis in the algae and can be passed to the host tissue for quenching [51]. Once produced, ROS have detrimental effects on the cell, including nucleic acid damage, lipid peroxidation of polyunsaturated fatty acids, protein oxidation, and the disruption of some signal transduction pathways [50,52]. It is therefore important for organisms to have systems that remove these radicals from their cells.

### 3.2. Examples of Oxidative Defense Compounds

Oxidant defense systems can be broadly categorized as either enzymatic—such as peroxiredoxins and thioredoxins—or non-enzymatic, including compounds like glutathione [53]. These systems are widespread across the animal kingdom, and are also present in Cnidaria, the evolutionarily ancient lineage that includes Medusozoa. Cnidaria diverged from Bilateria approximately 685–715 million years ago [54], and many of their oxidative defense mechanisms are conserved across more evolutionarily complex groups. For example, coelenterazine, a key antioxidant and bioluminescent molecule, is not only found in Cnidarians but also in squid (Cephalopoda), brittle stars (Ophiuroidea), and at least seven other marine phyla [55]. Although coelenterazine is present in Medusozoa, it cannot be synthesized by these organisms and must instead be acquired through diet [56]. In contrast, glutathione can be synthesized endogenously, as evidenced by increased levels in *Aurelia aurita* tissue following exposure to copper ions [57]. This molecule is also common across bacteria and eukaryotes more broadly [58]. The frequent blooms of Medusozoa and the simplicity of sample processing (e.g., freeze-drying) [59,60,61,62] have made them attractive candidates for antioxidant research. As a result, a growing number of oxidant defense systems have been described in Medusozoa, some of which appear taxon-specific while others are more universally distributed across eukaryotes (Table 1).

Many molecules involved with bioluminescence also have a role in oxidative defense. One of the theories for the evolutionary origin of bioluminescence is selection for bioluminescence from these oxidative defense pathways as organisms descended into more anoxic, less UV irradiated environments [64,71]. Coelenterazine, the primary luciferin found in Cnidaria, as well as eight other mainly marine phyla [55], shows an antioxidative effect against both singlet oxygen and superoxide anions, with rates similar to that of ascorbic acid [64]. It can both act as an oxidative defense in its own right, as well as an acceptor of radicals created during the reactions of other proteins with ROS [64]. In many Hydrozoans, light produced by coelenterazine is transferred to a fluorescent protein that changes the emitted fluorescence color from the blue of the photoprotein in vitro to a green, caused by the aptly named Green Fluorescent Protein (GFP) [72]. GFP has an enzymatic effect similar to superoxide dismutases (SOD), a group of enzymes that catalyze a reaction turning the superoxide anion into oxygen and hydrogen peroxide [73]. At low concentrations of O_2_^−^, quenching occurs with no structural effects to GFP and no effect on fluorescent ability. At high concentrations, structural changes occur that inhibit the protein’s ability to quench O_2_^−^, but do not significantly change bioluminescent ability [63]. Furthermore, during the photoactivation of GFP, singlet oxygen radicals will be produced [74].

A novel oxidation defense system was discovered in the scyphomedusa *Aurelia aurita*, whereby iodine ions taken in from the surrounding seawater react with ROS, especially hydrogen peroxide, to form elemental iodine [53]. This elemental iodine then further reacts with various phenols, mainly the amino acid tyrosine, to produce iodotyrosines such as monoiodotyrosines, diiodotyrosine, and thyroxin. These are considerably more membrane permeable than the iodine, enabling them to diffuse out of the cell and sacrificially remove radicals [53]. This system is intimately connected with the strobilation of some Scyphozoans, as iodine can act as a strobilation factor [53,75,76], although this may mean this oxidative defense system could be restricted to only the Scyphozoans that use iodine as a strobilation factor. Iodine and tyrosine are found in a wide variety of animals, with both required for the formation of thyroid hormones in vertebrates [77]. They may provide some form of oxidative defense in higher organisms, but the efficacy of this system may hinge on the ability of the organism to eliminate the iodotyrosines after they are created. Further research into the distribution and variability of this system across the Scyphozoa—and potentially beyond—could shed light on the evolutionary links between oxidative defense and life history regulation, and may also reveal novel biochemical pathways with relevance to both marine physiology and iodine metabolism in other taxa.

The compounds that are unique to Medusozoa are all genetic products [68,69,78]. Many of them may in fact be unique to the specific species they were found in, as the wider distribution of these proteins is yet unknown. Some show similarity with a variety of gene families found across the animal and plant kingdoms. HvAPX1 is a peroxidase, showing the most similarity to plant ascorbate peroxidases, and may have been introduced into the *Hydra* genome via horizontal gene transfer from a symbiotic algae species [66]. CcPrx4 is a peroxiredoxin, a subset of peroxidase enzymes that uses cysteine residues to catalyze the breakdown of hydrogen peroxide [68,79]. Finally, CcSOD1 is a copper- and zinc-based SOD, containing many active sites known to control protein function in these SOD enzymes by interaction with the metal ions [69]. In contrast with those compounds that fall into gene or protein families, SmP90, *ppod1*, and *ppod2* showed no significant similarity with other oxidant defense proteins [70,78]. Both *ppod1* and *ppod2* were found in multiple species of *Hydra*, and can also be used as molecular markers for foot differentiation in some species as they are specific to their basal disc [78]. Further study into these genes could expose more oxidant defense gene families in both Medusozoa and wider taxa, leading to a deeper understanding of how these simple organisms protect themselves, as well as potentially revealing evolutionary links to oxidant defense systems in higher organisms.

### 3.3. Distribution of Oxidative Defense Compounds

The research currently available on the distribution of oxidative defense compounds is not comprehensive. It has mainly been performed under the lens of biochemistry, and therefore the majority of studies do not isolate exact compounds, instead focusing on the activity of Medusozoan extracts as a whole, or broad fractions of the whole specimen’s proteome [60,62,80,81,82,83,84,85,86]. There is little focus to explain the reasoning behind the distribution of these compounds in an ecological sense. Since the research is all biochemically focused, it has all been done in vitro, leading to complications when the same effects are assumed to occur in vivo [87], especially when there is no data on the in vivo oxidant defensive activity in Medusozoa. Additionally, the studies were only carried out in Scyphozoa, mainly order Rhizostomeae, potentially due to ease of access from frequent blooms [60,88] and the current edible Scyphozoan market [89,90,91], providing a narrow and biased dataset when attempting to consider the entirety of the Medusozoa. Even with this issue of a biased dataset, the various assays for antioxidant activity give non-comparable results, either between different assays or the same assay between different laboratories [87,92]. Due to all these issues, we can draw no direct comparison from this data to its effect in vivo, and thus any comparisons we draw will strictly be qualitative.

Table 2 contains the studies that have analyzed the antioxidant activity of both the umbrella and oral arm of the same Medusozoan in the same study, all of which have been performed on Scyphozoans. A comparison between the oral arm and umbrella has been utilized as fractions analyzed and solvents used for extraction varied across studies so that a direct inter-study comparison is not feasible. Because of the small number of studies done in this area it is difficult to draw any conclusions from this data as the only two species with multiple studies about them are *Aurelia coerulea* [59,61] and *Cassiopea andromeda* [93,94]. The studies on *Aurelia coerulea* agree that there is an increased presence of antioxidant compounds in the oral arms and that there is an enrichment of phenols and proteins in the oral arms. In comparison, the studies on *Cassiopea andromeda* both agree that phenols and proteins are enriched in the oral arms compared to the umbrella, and yet there is no difference between the antioxidant ability of oral arm and umbrella tissue. For the majority of reported species, both phenols and proteins are enriched in the oral arms compared to the umbrella, but this does not always correlate with an increase in antioxidant activity. No correlations can be brought by looking at higher taxonomic levels either, as all the species except *Aurelia coerulea* are in family Rhizostomeae [95] and provide no comprehensive agreement across any of the three variables. Further research into this field may enable the reason for this distribution to be discovered and may provide crucial information for the commercialization of antioxidant compound extraction from Medusozoans.

## 4. Antimicrobial Peptides

The marine environment is home to a high density of bacterial populations, with an average of 10^5^–10^6^ bacteria per milliliter of seawater [97]. For this reason, Medusozoa require an immune system that is capable of preventing infection. Lacking an adaptive immune system, phagocytes, or any impermeable barriers [98,99,100], Medusozoa rely on their innate immune system and antimicrobial peptides to prevent infection. Many pathogenic infections of corals (Anthozoa) are known [101,102], while limited information is available about pathogens in Medusozoa, other than as potential vectors of infection for other organisms [103,104]. Nevertheless, protection from the dense microbial community of seawater is imperative for survival.

Antimicrobial peptides (AMPs) are a broad category of molecules with efficacies against a range of bacteria, fungi, and viruses. It is a comprehensive term that includes molecules with a wide range of mechanisms of action and structures, but overall, they represent the primary defense systems for multicellular organisms [105]. AMPs are a well-studied aspect of Medusozoan biology, with reviews already encompassing their identification and efficacy [106,107,108]. Here we aim to provide the updated available information by merging the existing reviews with new findings and providing an overview of AMPs, their distribution, and their efficacy in Medusozoa.

The majority of AMP research has been conducted on the Hydrozoa, specifically the genus *Hydra*, with a restricted number of studies focusing on Scyphozoa and the other classes of Medusozoa. *Hydra* are small freshwater Cnidarians in the class Hydrozoa that have been used as a model organism in a number of biological studies for years [109], including for host–microbial interactions [110]. A variety of taxon-specific antimicrobial peptides have been discovered from *Hydra* [106,108], making them, and the wider Medusozoa, an intriguing area of research for novel antimicrobials.

### 4.1. Efficacy of AMPs

Mariottini and Grice [106] provide an overview of almost all AMPs derived from Medusozoa, while Klimovich and Bosch [111] provide an insight into AMPs using ‘omics’ techniques and how this approach may inspire further research. Because of existing reviews on this topic, we will not go into detail on the efficacy to avoid redundancy. However, we provide an overview of the activity of the totality of Medusozoa (Table S1) by expanding the original table by Mariottini and Grice [106].

As we already mentioned above for antioxidants, different methods have been used for studies in vitro and in vivo. In animal models other than Cnidaria, there is a strong linear relationship between MIC and MBC, and in vivo antimicrobial activity [112], where in vitro effects translate well to those in vivo [113]. Conversely, in vivo data for Medusozoa are not available to corroborate or find a relationship with in vitro data (Table S1).

AMPs from *Hydra* show a wide range of efficacies against a variety of both Gram- positive and negative bacteria, including resistant strains such as methicillin resistant *Staphylococcus aureus*, or extended-spectrum beta-lactamase producing *Escherichia coli* [114]. Arminin-1a, isolated from *Hydra vulgaris*, is a potent AMP that has no sequence homology to any known AMP [115]. With high activity against a range of pathogens, it may be a template for a new group of antimicrobial drugs [116]. The list of AMPs isolated from Scyphozoa is a much shorter one, with aurelin being the only peptide so far isolated from the tissues of a Scyphozoan [117], albeit only showing moderate antimicrobial activity. Again, it shares no structural homology to known AMPs, but shares some similarities with defensins, a broad group of AMPs that all contain six disulfide-paired cysteines [118]. These compounds have mainly been tested against human pathogens for the sake of pharmaceutical discovery, but further research could aid in understanding the antimicrobial activity of these compounds against Cnidarian pathogens and their efficacy in in vivo protection.

### 4.2. Spatial Distribution of AMPs

#### 4.2.1. Hydrozoa

In terms of the distribution between body parts, the highest density of AMP expression in *Hydra* is found in the distal foot and hypostome (head) regions, as the spatial distribution of AMPs is dictated mainly by the distribution of microbiologically active neurons [119]. Both RFamideIII and NDA-1 are expressed in neurons found only in the hypostome and foot region, with NDA-1 limited to the far distal hypostome and base of the tentacles. RFamideIII has a slightly more expansive distribution, as it can be found throughout the tentacles, although it is not expressed in the most extreme region of the foot [119]. Hym-357 and Hym-370 have a wider distribution than the previous two peptides, being expressed in the body column as well as the foot and hypostome. Hym-370 is expressed more densely, and Hym-357 shows a lack of expression in the most extreme region of the foot [119]. Expression of these four peptides is limited to the distal part of sensory neurons that face the outer mucus layer, implying that they are secreted into the glycocalyx and mucus layer [109]. These peptides aid in controlling the microbiome found in the glycocalyx layers [120,121] and prevent invasion from non-symbiotic microbes.

With non-neuronally expressed AMPs, the distribution depends predominantly on the tissue layers. The endodermal epithelium is where the greatest concentration of AMPs is expressed, as the epithelium around the gastral cavity is the most at risk of infection due to the regular intake of food containing a wide range of potentially harmful microbes [122]. Arminin-1a, Hydramacin-1, and Periculin-1 are all expressed in the endodermal epithelium, with Periculin-1 also being expressed in some endodermal interstitial cells [100,115]. Kazal-2 is expressed in gland cells in the endodermal epithelium along the whole-body column except for the extreme hypostomal and foot ends [123]. It is then secreted into the gastral cavity upon food intake to prevent microbial overgrowth during digestion. AMPs expressed in the ectoderm are mainly those previously mentioned that are neuronally expressed and secreted into the glycocalyx [111].

#### 4.2.2. Scyphozoa

In terms of the distribution in the body tissues, although a specific peptide was not isolated, lysozyme-like activity was highest in the oral arms of *Aurelia coerulea*, intermediate in the umbrella, and lowest in the mucus [59]. This trend is the same as shown by antioxidant activity in the same species [59], although the drivers of both trends are currently unknown and therefore further studies are required to unravel the triggers of such trends and whether they are common to other members of the Scyphozoa.

Due to the low number of AMPs isolated from Scyphozoa, there is little data available on the body layer distribution of AMPs in this class. The only AMP that has been isolated from the body tissue of a Scyphozoan, aurelin, was isolated from the mesoglea [117]. An AMP has been described from *Cassiopea xamachana* showing homology with aurelin that shows putative effects against *Klebsiella pneumoniae*, but has not been synthesized or tested ex silico, nor has its distribution been identified [124].

### 4.3. Temporal Variability in AMP Distribution

#### 4.3.1. Hydra

**Figure 2:**
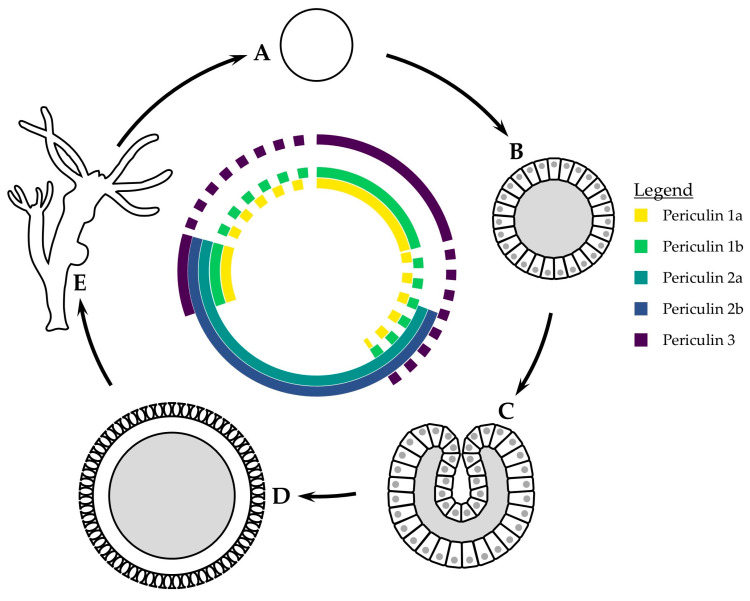
Variation in expression of periculins over the Hydra life cycle. ‘A’ egg; ‘B’ blastula; ‘C’ gastrula; ‘D’ cuticle stage; ‘E’ adult polyp. Stage images based on electron micrographs from Martin, et al. [129].

External eggs exposed to the marine environment require a defense system against microbial colonization. Antimicrobial defenses in eggs have been found across a wide number of marine invertebrates [125,126,127] and in other Cnidarians [106]. The eggs of *Hydra* are protected by maternally expressed periculins in their early development, until the developing embryo begins to synthesize its own specific forms of AMPs and the maternal line of periculins is downregulated. During embryogenesis, maternal periculins are the dominant AMPs. Periculins 1a, 1b, and 3 are exclusive to the female germline, and are strongly expressed in interstitial cells in the ectoderm of adult polyps. Additionally, they are expressed in the late stage of oogenesis until the blastula is formed, with expression stopping completely after the formation of the gastrula (Figure 2) [128]. After the mid-blastula transition, periculins 2a and 2b are expressed in blastomeres of the outer epithelial layer (Figure 2) [128]. After the first cleavage, periculins are found uniformly over the embryo surface. Once the cuticle has formed (an intermediate dormancy period in *Hydra* embryogenesis [129]), periculins are found in patches on the outside of the cuticle [128].

#### 4.3.2. Scyphozoa

In Scyphozoans, only the extract from eggs of *Rhizostoma pulmo* has been reported as showing lysozyme-like effects against the cell walls of a Gram-positive bacteria, *Micrococcus luteum* [130]. The exact peptide performing this activity has not yet been isolated.

## 5. Endobionts

In Cnidaria, several symbioses are well known, including the intimate relationship between dinoflagellate algae and corals [131], or clownfish and anemones [132]. Despite the fact that Medusozoa also have symbiotic relationship with dinoflagellates as well as other organisms [133,134,135,136,137,138,139,140,141,142], this aspect of their biology remains less known compared with the abovementioned symbioses of Anthozoa, even when microbial symbioses in Medusozoa are important for their survival [143]. Aposymbiotic individuals often have remarkably lower fitness than their counterparts with the native microbiome [143,144,145,146]; but once aposymbiotic polyps are recolonized by their native microbiome, they will regain this lost fitness [143]. In addition to modulating survival and reproduction, endobionts can have defensive roles, with some synthesizing AMPs [147] or multi-bacterial microbiomes protecting against fungal infection [121].

Intrinsic genetic factors may have significant influence on the colonizing microbiota, as amongst Scyphozoa of the same species from varying localities, the microbial community composition remains distinct over a decade of growth in identical ambient conditions [138]. In *Hydra*, the maintenance of a distinct community has been observed over 30 years of growth in a laboratory [148]. The microbial community of these organisms is usually very different to that of the surrounding seawater, although with certain body parts hosting communities more comparable to their environment [138]. Since numerous species contain host-specific microbes [135,138,140], this may indicate co-evolution between the host and the microbiome, although at present it is unclear what role these microbes may play in the host organism. In the natural environment, interactions between intrinsic genetic factors and environmental variables likely dictate the final microbiome.

### 5.1. Host Benefits of Endosymbionts

Some endosymbiotic fungi [147,149] and bacteria [150] produce antimicrobial compounds. These may aid in modulating the microbiome to reduce competition for the AMP-producing endobiont, or provide increased AMP production for the host to maintain microbial load at an appropriate level. Interactions between endobiotic microbes can help in preventing fungal infections, with the highest level or protection being offered when a complex microbiome is present [121]. These microbes may be a promising source of antimicrobials from Medusozoa with simpler cultivation methods than farming the hosts.

Bioluminescence is exceptionally prevalent within specific classes of Medusozoa [55,151,152]. However, some species harbor species of bioluminescent bacteria instead of having intrinsic bioluminescence [153,154], or simply to supplement their own luminescence [155]. These bioluminescent bacteria are all *Vibrio* spp., a genus of bacteria that feeds on chitin and are therefore found in chitin dense areas of the Hydrozoan structural organs. At this current time, it is difficult to say what purpose this bioluminescence may have, and if it is an example of symbiosis, like *Vibrio fischeri* and *Euprymna scolopes* [156], or infection found across a range of marine vertebrates and invertebrates [155]. Further study into this area could bring to light the specifics of how these bacteria interact with Medusozoa, and potentially whether Medusozoans may act as a vector for this marine pathogen.

Some of the microbiota discovered from Medusozoa produce highly toxic compounds. Samples of both Hydrozoa and Scyphozoa contained a large number of host-specific bacteria, many of which were close phylogenetically to bacteria that produce a variety of cytotoxic, hemolytic, septicemic, and necrotic toxins [140,157]. Among them, *Pseudoalteromonas tetraodonis* group one produces tetrodotoxin, a potent neurotoxin [158]. The presence of these toxins may help deter predators, and seem not to harm the host, providing a defensive benefit. Since the microbiome of each species is unique and varies considerably, additional research on a wider range of Medusozoa will allow identification of potential toxins and evaluate their potential in biotechnological applications. Conversely, these toxins may pose a problem for the expansion of the edible jellyfish market into new species, as some taxa may require additional processing to remove additional non-host toxins.

### 5.2. Spatial Distribution of Endobionts

#### 5.2.1. Hydrozoa

Although *Hydra* may dominate research into AMPs within Medusozoa, studies on the distribution of their microbiome are scarce. Aggregates of bacteria form in the tips of some Hydrozoans’ tentacles, although the role of these aggregates is unknown [140,157]. Additionally, the microbiome in the endoderm surrounding the gastral cavity is not stable [122], likely because this area has high concentrations of various AMPs.

#### 5.2.2. Scyphozoa

Bacterial communities in the mucus show higher similarity to those found in the surrounding seawater than in other body parts, albeit with higher diversity than the water [138]. This similarity is most likely due to the influence of seawater upon the mucus, which leads to a highly variable microbial community composition not found in other body compartments. Mucus also houses the highest abundance of bacteria found in body compartments of Scyphozoa [133], potentially due to it being a reservoir for easily accessible nutrient sources [133].

The oral arms and umbrella tend to have similar bacterial community composition [133,159]. Even still, the oral arms usually show a higher bacterial diversity than the umbrella, although the opposite trend has been reported [160]. However, in this case the umbrella and gastral cavity were combined, which may have inflated richness estimates for the umbrella. In *Aurelia solida*, the umbrella and oral arms share a bacterial community mostly within Alphaproteobacteria (mainly Roseobacteraceae—genera *Phaeobacter* and *Rugeria*) and Gammaproteobacteria (mainly Vibrionaceae—genus *Vibrio*) [159], whereas *Rhizostoma pulmo*, the umbrella, and oral arms mainly contain Mollicutes, majority genera *Spiroplasma*, and *Mycoplasma* [133].

The gastral cavity of a Scyphozoan has the most distinct microbial community compared to the surrounding seawater [138] and the other body parts [159]. In the gastral cavity, the microbial diversity tends to be lower and dominated by a small number of specific species [159,161,162]. Nevertheless, in some gastral cavities’, the bacterial diversity was similar to that of the umbrella, albeit still with a distinct community composition [159]. The microbial community of the gastral cavity in Scyphozoa also differs between species. *Cotylorhiza tuberculata* was dominated by genera *Spiroplasma* (Mollicutes), *Thalassospira* (Alphaproteobacteria), *Tenacibaculum* (Flavobacteriia), and *Synechococcus* (Cyanophyceae), constituting ~95% of the observed diversity [162]. On top of these there were multiple novel species identified [161], although ‘*Candidatus* Syngnamydia medusae’ has recently been isolated from a protist [163]. This finding suggests that these novel species may not be unique to *Cotylorhiza tuberculata*, but simply not known in their other hosts. *Aurelia aurita* also had a Mollicutes species as the foremost member of the gastral cavity microbiome, but an unknown *Mycoplasma* sp. [138]. Conversely, the gastral cavity of *Aurelia solida* is dominated by the Betaproteobacteria, mainly Burkholderiaceae, a bacteria family known to be antibiotic resistant [159]. If the gastral cavity of Scyphozoa follows the trend of AMP presence of *Hydra*, this may provide an advantage to the Burkholderiaceae, but this has not been observed in any Scyphozoan species to date. Due to the number of unique species and the distinctness of the gastral cavity microbiome, it poses a promising area for the identification of more novel species and potential products from those microbes.

The subumbrella of the Scyphozoa is home to the highest fungal diversity, with a small number also being isolated from the tentacles [149]. No fungi were found on the exumbrella or gonad, areas that can contain diverse bacterial communities [133,159]. Since some bacteria found in *Hydra* show antifungal properties [121], similar strains in Scyphozoans may prevent fungal proliferation in areas rich in bacteria.

### 5.3. Temporal Variability of Endobionts

#### 5.3.1. Hydrozoa

Although bacterial diversity decreases with age post-hatching, bacterial abundance is not correlated with age. Diversity shows a distinct rise around four weeks post-hatching, where the microbiome is similar to that of the adult polyp, after which the microbiome shifts before building up to the final adult community composition [164].

#### 5.3.2. Scyphozoa

Overall, Scyphozoans have two major life stages—an asexual benthic polyp, and a sexual pelagic medusa [165], with the exception of some species which only have either one stage or the other [165]. The transition between benthic and pelagic stages is highly dependent on the microbiome, with aposymbiotic individuals often having remarkably lower settlement, strobilation, and ephyra production rates [143,144,145]. Despite this dependence, the net change in microbial richness is different according to the species of the host. Different species lose, gain [160], or maintain an unchanged [138] microbial richness across their life stages. Independent of any diversity shift, the specific operational taxonomic units (OTUs—clusters of genetic sequences with 97% similarity or more [166]), or species, that make up an individual’s microbiome can be variable, leading to each life stage being characterized by a specific microbiome [138]. Major changes in the body, such as strobilation or excystation, cause significant changes to the microbial community structure [144,167].

Between the stages, a subset of the microbial community forms a stable community in time, as observed in *Chrysaora plocamia*, where about 15% of the OTUs were maintained from polyp through to excyst [167]. Many core microbes were involved in major elemental cycles, potentially playing an important role in nutrient acquisition for the individual. *Aurelia aurita* shows this pattern only in part, with some bacterial OTUs showing vertical transmission between life stage, such as *Crenothrix* (Gammaproteobacteria) being found in polyps through to juvenile medusae [138]. While a core community has not been reported for *A. aurita* so far, 16 OTUs are shared between *C. plocamia*’s core community and *A. aurita* life stages [167]. The definition of core microbiomes across a greater number of Scyphozoan species and their role within the metabolic pathways within the organisms will help to shed light on the biology of this group of marine organisms and suggest potential applications in biotechnology.

## 6. Conclusions

While review papers have summarized the knowledge currently available for Anthozoa, a state-of-the-art review about the ecological knowledge and potential biotechnological applications of defensive systems other than toxins in Medusozoa was lacking. In the present review, we attempted to fill this gap by collecting the available literature and organizing the data into the four topics which received attention in previous research efforts. Our goal was to bridge ecological knowledge with biotechnology in order to improve our understanding of this group of organisms often massively present in the marine ecosystem and suggest potential fields where their biomass may be exploited within the context of sustainable resource use.

The greater amount of data available for antimicrobial peptides compared to antioxidants, photoprotective, and endosymbiotic-derived compounds suggest that this line of research may lead to major exploitation of Medusozoa. However, some organisms, considered as models, have been studied in detail, while knowledge of defensive systems in other Medusozoa is still very limited or totally absent. However, the close phylogenetic relationship within Medusozoa suggests that the same patterns/strategies may be shared by multiple organisms within the group. Therefore, an integrated approach to the study of chemical defenses will aid in better understanding the biology and ecology of this group of organisms, but also support the exploitation of Medusozoan biomass for biotechnological applications.

## Figures and Tables

**Table 1:** Oxidative defense systems with specific enzymes/compounds and their associated quenched radicals found in Medusozoa, according to the available literature.

Defense System	Quenched Radical	Species	Unique to Medusozoa	Reference
GFP	O_2_^−^	-	No	[63]
Coelenterazine	O_2_^−^ and ^1^O_2_^−^	-	No	[64]
Glutathione	Various	-	No	[57]
Ovothiol	H_2_O_2_	-	No	[65]
Iodide—Tyrosine	H_2_O_2_	*Aurelia aurita*	Unknown	[53]
HvAPX1	H_2_O_2_	*Hydra viridissima*	Yes	[66]
ppod1	H_2_O_2_	*Hydra vulgaris*	Yes	[67]
ppod2	H_2_O_2_	*H. vulgaris*	Yes	[67]
CcPrx4	H_2_O_2_	*Cyanea capillata*	Yes	[68]
CcSOD1	O_2_^−^	*C. capillata*	Yes	[69]
SmP90	O_2_^−^	*Stomolophus meleagris*	Yes	[70]

**Table 2:** Comparison of antioxidant activities, phenol, and protein contents between the oral arms and the umbrella of various scyphomedusae (Cnidaria, Scyphozoa) divided by taxonomic order, with ‘Higher’ indicating a higher activity/content in the oral arms compared with the umbrella. All comparisons in antioxidant activity were performed using values normalized for protein content.

Order	Species	Antioxidant Activity	Phenols	Proteins	Reference
Semaeostomeae	*Aurelia coerulea*	Higher	Higher	Higher	[59]
		Higher	Higher	Higher	[61]
Rhizostomeae	*Cotylorhiza tuberculata*	Higher	Higher	Higher	[61]
	*Cassiopea andromeda*	No Difference	Higher	Higher	[93]
		No Difference	Higher	Higher	[94]
	*Catostylus tagi*	No Difference	–	Higher	[96]
	*Rhizostoma pulmo*	No Difference	No Difference	No Difference	[61]

## Data Availability

Not applicable.

## Supplementary Materials

The following supporting information can be downloaded at: https://www.mdpi.com/article/10.3390/md23060229/s1, Table S1: Antimicrobial peptides discovered and tested from Medusozoa and their endobionts; Table S2: Due to discrepancies in reported methods for calculating both MIC and MBC, a summary method for each reference will be included here. We advise to check the original reference for specific details [168,169,170].
