# Supplementary material for: Chemical Defenses in Medusozoa
Source: Mar Drugs. 2025 May 28;23(6):229. doi: 10.3390/md23060229 (PMC12194522; doi:10.3390/md23060229)
Supplement: Supplementary file 1 [file marinedrugs-23-00229-s001.zip › marinedrugs-3644977-supplementary.pdf]

Table S1. Antimicrobial peptides discovered and tested from Medusozoa and their endobionts. '+' = Gram Positive Bacterium, '-' = Gram Negative Bacterium, '+*' = Multi-drug Resistant Gram Positive Bacterium, '-*' = Multi-drug Resistant Gram Negative Bacterium, '~' = Fungus. 'MBC' = Minimum Bactericidal Concentration, >99.9% killing of bacteria in a microdilution assay. 'MIC' = Minimum Inhibitory Concentration

Species	Name	Sensitive Microbe	MBC µM	MIC µM	LD90 µM	Reference
Aurelia aurita	Aurelin (Recombinant)	*Bacillus megaterium* +		10		[1]
		Micrococcus luteus +		40		[1]
	Aurelin	*Listeria monocytogenes* +		5.27		[2]
		Escherichia coli -		1.78		[2]
Hydra vulgaris	RFamide III (Synthesized)	*Bacillus megaterium* +		0.7		[3]
		Bacillus subtilis +		>500		[3]
		Trichococcus collinsii +		0.5-0.9		[3]
		Trichococcus pasteurii +		0.9		[3]
		Acinetobacter sp. -		1.8		[3]
		Curvibacter sp. -		58.2		[3]
		Escherichia coli -		461		[3]
		Pseudomonas sp. -		58.2		[3]
	Hym-121	*Bacillus megaterium* +		6.3		[4]
		Acidovorax sp. -		1.5		[4]
		Curvibacter sp. -		25		[4]
		Duganella sp. -		12.5		[4]
		Escherichia coli -		0.2		[4]
		Undibacterium sp. -		>100		[4]
	Hym-357 (Synthesized)	*Bacillus megaterium* +		4.4		[3]
		Bacillus megaterium +		4		[4]
		Bacillus subtilis +		22		[3]
		Trichococcus collinsii +		0.3		[3]
		Trichococcus pasteurii +		1.4		[3]
		Acidovorax sp. -		>100		[4]
		Acinetobacter sp. -		11		[3]
		Curvibacter sp. -		>500		[3]
		Curvibacter sp. -		>100		[4]
		Duganella sp. -		>100		[4]
		Escherichia coli -		>500		[3]
		Escherichia coli -		>100		[4]
		Pseudomonas sp. -		>500		[3]
		Undibacterium sp. -		>100		[4]

Hydra vulgaris	Hym-370 (Synthesized)	*Bacillus megaterium* +		1.8		[3]
		Bacillus megaterium +		2		[4]
		Bacillus subtilis +		4.5-9.0		[3]
		Trichococcus collinsii +		0.3		[3]
		Trichococcus pasteurii +		0.6		[3]
		Acidovorax sp. -		50		[4]
		Acinetobacter sp. -		2.2		[3]
		Curvibacter sp. -		>500		[3]
		Curvibacter sp. -		100		[4]
		Duganella sp. -		>100		[4]
		Escherichia coli -		72		[3]
		Escherichia coli -		25		[4]
		Pseudomonas sp. -		143.6		[3]
		Undibacterium sp. -		>100		[4]
	Periculin-1 (Recombinant)	*Bacillus megaterium* +			0.2-0.4	[5]
	NDA-1 (Recombinant)	*Bacillus megaterium* +		0.4		[3]
		Bacillus subtilis +		5.8		[3]
		Staphylococcus aureus +		23.1		[3]
		Trichococcus collinsii +		0.4-0.9		[3]
		Trichococcus pasteurii +		0.9		[3]
		Acinetobacter sp. -		0.7		[3]
		Curvibacter sp. -		0.4		[3]
		Escherichia coli -		>14		[3]
		Pseudomonas sp. -		>20.9		[3]
	kazal2 Domains (Recombinant)	*Staphylococcus aureus* +	38	38		[6]
		Staphylococcus aureus +	36	36		[6]
		Staphylococcus aureus +	33	33		[6]
	kazal2	*Staphylococcus aureus* +		0.7-0.8		[6]
	Hydramacin-1	*Enterococcus faecalis VRE* +*	>14.3		7.1	[7]
		Staphylococcus aureus MRSA +*	>14.3		>14.3	[7]
		Bacillus megaterium +	0.2		0.1	[5]
		Enterococcus faecalis +	14.3		0.9	[5]
		Staphylococcus aureus +	>14.3		>14.3	[7]
		Staphylococcus aureus +	>14.3		14.3	[5]
		Staphylococcus epidermidis +	>14.3		>14.3	[5]
		Staphylococcus haemolyticus +	1.8		0.9	[7]
		Staphylococcus hominis +	>14.3		3.6	[5]

		Streptococcus pneumoniae +	>14.3	>14.3	[5]
		Streptococcus pyogenes +	>14.3	7.1	[7]
		Escherichia coli ESBL -*	3.6	0.9	[7]
		Escherichia coli ESBL -*	0.4	0.4	[5]
		Escherichia coli ESBL -*	0.4	0.2	[5]
		Escherichia coli ESBL -*	0.9	0.4	[5]
		Escherichia coli ESBL -*	0.9	0.2	[5]
		Escherichia coli ESBL -*	0.9	0.2	[5]
		Klebsiella oxytoca ESBL -*	7.1	0.9	[7]
		Klebsiella oxytoca ESBL -*	3.6	0.9	[7]
		Klebsiella oxytoca ESBL -*	0.9	0.2	[5]
		Klebsiella oxytoca ESBL -*	0.9	0.4	[5]
		Klebsiella oxytoca ESBL -*	0.9	0.4	[5]
		Klebsiella oxytoca ESBL -*	0.9	0.4	[5]
		Klebsiella pneumoniae ESBL -*	3.6	0.9	[7]
		Klebsiella pneumoniae ESBL -*	3.6	0.9	[5]
		Klebsiella pneumoniae ESBL -*	1.8	0.4	[5]
		Klebsiella pneumoniae ESBL -*	0.9	0.4	[5]
Hydra vulgaris	Hydramacin-1	*Acinetobacter naumannii* -	7.1	1.8	[7]
		Burkholderia cepacia -	>14.3	>14.3	[7]
		Burkholderia cepacia -	>14.3	>14.3	[7]
		Burkholderia cepacia -	>14.3	>14.3	[7]
		Citrobacter freundii -	7.1	0.9	[7]
		Citrobacter freundii -	0.9	0.5	[7]
		Citrobacter freundii -	0.9	0.2	[5]
		Enterobacter cloacae -	>14.3	0.9	[7]
		Enterobacter cloacae -	0.9	0.5	[7]
		Enterobacter cloacae -	1.8	0.4	[5]
		Escherichia coli -	0.9	0.2	[7]
		Escherichia coli -	0.9	0.4	[5]
		Escherichia coli -	0.4	0.2	[5]
		Escherichia coli -	7.1	0.4	[5]
		Klebsiella oxytoca -	0.9	0.5	[7]
		Klebsiella pneumoniae -	0.9	0.5	[7]
		Klebsiella pneumoniae -	0.9	0.4	[5]
		Proteus mirabilis -	14.3	0.9	[7]
		Proteus vulgaris -	>14.3	3.6	[7]
		Providencia rettgeri -	>14.3	>14.3	[7]

		Pseudomonas aeruginosa -	>14.3	14.3	[7]
		Pseudomonas aeruginosa -	>14.3	>14.3	[5]
		Salmonella typhimurium -	0.9	0.5	[7]
		Salmonella typhimurium -	0.9	0.5	[7]
		Salmonella typhimurium -	0.9	0.2	[5]
		Serratia marcescens -	>14.3	3.6	[7]
	Hydramacin-1	*Serratia marcescens* -	>14.3	14.3	[7]
		Serratia marcescens -	>14.3	1.8	[7]
		Yersinia enterocolitica -	0.9	0.2	[7]
		Yersinia enterocolitica -	0.4	0.2	[5]
		Candida albicans ~	>14.3	>14.3	[5]
		Candida albicans ~	>14.3	>14.3	[5]
		Candida glabrata ~	>14.3	>14.3	[5]
		Enterococcus faecalis VRE +*	1.6	0.8	[8]
		Enterococcus faecalis VRE +*	0.4	0.2	[8]
Hydra vulgaris		*Enterococcus faecalis VRE* +*	0.8	0.2	[8]
		Enterococcus faecalis VRE +*	0.4	0.2	[8]
		Enterococcus faecalis VRE +*	0.4	0.2	[8]
		Staphylococcus aureus MRSA +*	0.4	0.2	[8]
		Staphylococcus aureus MRSA +*	0.4	0.2	[8]
		Staphylococcus aureus MRSA +*	0.8	0.2	[8]
	c-arminin-1a (Recombinant)	*Staphylococcus aureus MRSA* +*	0.8	0.2	[8]
		Staphylococcus aureus MRSA +*	0.4	0.2	[8]
		Bacillus megaterium +	0.1	0.01	[8]
		Staphylococcus aureus +	0.4	0.05	[8]
		Escherichia coli ESBL -*	0.4	0.1	[8]
		Escherichia coli ESBL -*	0.2	0.05	[8]
		Klebsiella pneumoniae ESBL -*	0.8	0.2	[8]
		Klebsiella pneumoniae ESBL -*	0.4	0.2	[8]
		Klebsiella pneumoniae ESBL -*	0.4	0.2	[8]
		Escherichia coli -	0.2	0.1	[8]
Paecilomyces variotii (*Nemopilema nomurai* endofungus)	Paecilocin A	*Staphylococcus aureus MRSA* +*	>152.7		[9]
		Staphylococcus aureus +	>152.7		[9]
		Vibrio parahaemolyticus MDR -*	>152.7		[9]
	Paecilocin B	*Staphylococcus aureus MRSA* +*	64.9		[9]
		Staphylococcus aureus +	16.2		[9]
		Vibrio parahaemolyticus MDR -*	>129.9		[9]

Paecilomyces variotii (*Nemopilema nomurai* endofungus)	Paecilocin C	*Staphylococcus aureus MRSA* +*	129.9	[9]
		Staphylococcus aureus +	64.9	[9]
		Vibrio parahaemolyticus MDR -*	>129.9	[9]
Streptomyces sp. (*Cassiopea xamachana* endobacterium)	Salinamide A	*Staphylococcus pyrogenes* +	3.93	[10]
		Streptococcus pneumoniae +	3.93	[10]
	Salinamide B	*Staphylococcus pyrogenes* +	1.89	[10]
		Streptococcus pneumoniae +	3.79	[10]

Table S2. Due to discrepancies in reported methods for calculating both MIC and MBC, a summary method for each reference will be included here. We advise to check the original reference for specific details.

Reference	Method for MIC	Method for MBC
[1]	Modified broth microdilution assay. Bacterial growth was evaluated with absorbance at 620nm. MIC was defined as the lowest concentration of the peptide that prevented visible bacterial growth after overnight incubation.	-
[2]	Radial diffusion assay by the agarose gel overlay technique. MIC was defined by "x" intersect of a regression line through zone diameters obtained from series of serially diluted peptide samples in radial diffusion assay.	-
[3]	Microdilution susceptibility assay. MIC was determined as the lowest serial dilution showing absence of a bacterial cell pellet.	-
[4]	Microdilution susceptibility assay. MIC was determined as the lowest serial dilution showing absence of a bacterial cell pellet.	-
[5]	-	MBC determined as >99.9% killing of bacteria from serial dilutions of the peptide
[6]	Microdilution susceptibility assay MIC defined as the peptide dilution where no bacterial sediments could be detected after incubation	MBC defined as the peptide dilution where no bacterial colonies formed when plated on LB-agar
[7]	-	MBC determined as >99.9% killing of bacteria from serial dilutions of the peptide
[8]	-	MBC determined as >99.9% killing of bacteria from serial dilutions of the peptide
[9]	Broth microdilution assay with the modified Mcfarland 0.5 standard. Turbidity was measured at 600nm. MIC was defined as the lowest concentration that inhibited more than 50% of visible [bacterial] growth after 72 h	-
[10]	No method is provided	-

Supplementary References

1. Shenkarev, Z.O.; Panteleev, P.V.; Balandin, S.V.; Gizatullina, A.K.; Altukhov, D.A.; Finkina, E.I.; Kokryakov, V.N.; Arseniev, A.S.; Ovchinnikova, T.V. Recombinant expression and solution structure of antimicrobial peptide aurelin from jellyfish *Aurelia aurita*. *Biochemical and Biophysical Research Communications* **2012**, *429*, 63-69, doi:https://doi.org/10.1016/j.bbrc.2012.10.092.
2. Ovchinnikova, T.V.; Balandin, S.V.; Aleshina, G.M.; Tagaev, A.A.; Leonova, Y.F.; Krasnodembsky, E.D.; Men'shenin, A.V.; Kokryakov, V.N. Aurelin, a novel antimicrobial peptide from jellyfish *Aurelia aurita* with structural features of defensins and channel-blocking toxins. *Biochemical and Biophysical Research Communications* **2006**, *348*, 514-523, doi:https://doi.org/10.1016/j.bbrc.2006.07.078.
3. Augustin, R.; Schröder, K.; Murillo Rincón, A.P.; Fraune, S.; Anton-Erxleben, F.; Herbst, E.-M.; Wittlieb, J.; Schwentner, M.; Grötzinger, J.; Wassenaar, T.M.; et al. A secreted antibacterial neuropeptide shapes the microbiome of *Hydra*. *Nature Communications* **2017**, *8*, 698, doi:10.1038/s41467-017-00625-1.
4. Klimovich, A.; Giacomello, S.; Björklund, Å.; Faure, L.; Kaucka, M.; Giez, C.; Murillo-Rincon, A.P.; Matt, A.-S.; Willoweit-Ohl, D.; Crupi, G.; et al. Prototypical pacemaker neurons interact with the resident microbiota. *Proceedings of the National Academy of Sciences* **2020**, *117*, 17854-17863, doi:doi:10.1073/pnas.1920469117.
5. Bosch, T.C.G.; Augustin, R.; Anton-Erxleben, F.; Fraune, S.; Hemmrich, G.; Zill, H.; Rosenstiel, P.; Jacobs, G.; Schreiber, S.; Leippe, M.; et al. Uncovering the evolutionary history of innate immunity: The simple metazoan *Hydra* uses epithelial cells for host defence. *Developmental & Comparative Immunology* **2009**, *33*, 559-569, doi:https://doi.org/10.1016/j.dci.2008.10.004.
6. Augustin, R.; Siebert, S.; Bosch, T.C.G. Identification of a kazal-type serine protease inhibitor with potent anti-staphylococcal activity as part of *Hydra*'s innate immune system. *Developmental & Comparative Immunology* **2009**, *33*, 830-837, doi:https://doi.org/10.1016/j.dci.2009.01.009.
7. Jung, S.; Dingley, A.J.; Augustin, R.; Anton-Erxleben, F.; Stanisak, M.; Gelhaus, C.; Gutsmann, T.; Hammer, M.U.; Podschun, R.; Bonvin, A.M.; et al. Hydramacin-1, structure and antibacterial activity of a protein from the basal metazoan *Hydra*. *J Biol Chem* **2009**, *284*, 1896-1905, doi:10.1074/jbc.M804713200.
8. Augustin, R.; Anton-Erxleben, F.; Jungnickel, S.; Hemmrich, G.; Spudy, B.; Podschun, R.; Bosch, T.C.G. Activity of the Novel Peptide Arminin against Multiresistant Human Pathogens Shows the Considerable Potential of Phylogenetically Ancient Organisms as Drug Sources. *Antimicrobial Agents and Chemotherapy* **2009**, *53*, 5245-5250, doi:10.1128/aac.00826-09.
9. Liu, J.; Li, F.; Kim, E.L.; Li, J.L.; Hong, J.; Bae, K.S.; Chung, H.Y.; Kim, H.S.; Jung, J.H. Antibacterial Polyketides from the Jellyfish-Derived Fungus *Paecilomyces variotii*. *Journal of Natural Products* **2011**, *74*, 1826-1829, doi:10.1021/np200350b.
10. Trischman, J.A.; Tapiolas, D.M.; Jensen, P.R.; Dwight, R.; Fenical, W.; McKee, T.C.; Ireland, C.M.; Stout, T.J.; Clardy, J. Salinamides A and B: anti-inflammatory depsipeptides from a marine streptomycete. *Journal of the American Chemical Society* **1994**, *116*, 757-758, doi:10.1021/ja00081a042.
